# Optimizing Nitrogen Fertilization and Irrigation Practices for Enhanced Winter Wheat Productivity in the North China Plain: A Meta-Analysis

**DOI:** 10.3390/plants14111686

**Published:** 2025-05-31

**Authors:** Donglin Wang, Shaobo Liu, Mengjing Guo, Yuhan Cheng, Longfei Shi, Jipo Li, Yongjie Yu, Siyu Wu, Qinge Dong, Jiankun Ge, Xuewen Gong

**Affiliations:** 1 College of Water Conservancy, North China University of Water Resources and Electric Power, Zhengzhou 450000, China; wangdonglin@ncwu.edu.cn (D.W.); guomengjing0417@163.com (M.G.); chengyvhan6714@163.com (Y.C.); shilongfei159@163.com (L.S.); lijipo@ncwu.edu.cn (J.L.); yuyongjie@ncwu.edu.cn (Y.Y.); gejiankun@ncwu.edu.cn (J.G.); gongxuewen@ncwu.edu.cn (X.G.); 2 School of Water Resources and Environment Engineering, Nanyang Normal University, Nanyang 473061, China; 3 Henan Key Laboratory of Water-Saving Agriculture, Zhengzhou 450000, China; 4 College of Water Resources and Civil Engineering, China Agricultural University, Beijing 100083, China; aoni0926@163.com; 5 Institute of Water-Saving Agriculture in Arid Areas of China (IWSA), Northwest A&F University, Yangling 712100, China; qgdong2014@nwafu.edu.cn

**Keywords:** winter wheat, irrigation practices, meta-analysis, nitrogen use efficiency (NUE), water use efficiency (WUE)

## Abstract

This study aimed to systematically evaluate the effects of different nitrogen application rates and irrigation practices on water-saving and yield enhancement in winter wheat production in the North China Plain (NCP) using a meta-analysis. By quantifying the impacts on crop yield, nitrogen use efficiency (NUE), and water use efficiency (WUE), the research provides a scientific basis for optimizing management practices in winter wheat production in this region. A comprehensive literature search was conducted across multiple databases, resulting in the inclusion of 94 eligible studies from 2018 to 2023. A random-effects model was employed to calculate the combined effect sizes, followed by subgroup and sensitivity analyses to further investigate the influence of nitrogen application rates, irrigation methods, and study regions on winter wheat production efficiency. The findings reveal that increasing nitrogen application rates and adopting deficit irrigation practices significantly improved winter wheat yield (combined effect size: 4.53 t·ha^−1^), NUE (43.29%), and WUE (0.013 t·ha^−1^·mm^−1^). The subgroup analysis further elucidated the critical roles of nitrogen application ratios, irrigation methods, and study regions in determining winter wheat production efficiency, while the sensitivity analysis confirmed the robustness of these findings, as the pooled effect sizes decreased by merely 0.69% and increased by 0.61% after excluding small-sample or highly biased studies, respectively. The above meta-analysis did not incorporate long-term field trials; hence, two-year field experiments with designed irrigation and organic–inorganic fertilizer treatments were conducted, which provided further validation for the meta-analysis. Under short-term conditions (excluding CO_2_ effects), we observed that chemical fertilizer exhibited a measurable inhibitory effect on crop water uptake and optimal water–fertilizer management was achieved with a 7:3 inorganic–organic fertilizer ratio combined with 450 m^3^·ha^−1^ irrigation. This study demonstrates the effectiveness of optimizing nitrogen fertilization and irrigation management in enhancing winter wheat yield and resource utilization efficiency. The findings offer actionable insights for sustainable agricultural practices in the NCP and similar regions, contributing to improved crop productivity and resource conservation.

## 1. Introduction

The North China Plain (NCP) is one of China’s most critical grain production bases, serving as a cornerstone of the nation’s food security. Winter wheat, as a staple crop in this region, plays a vital role in ensuring stable grain supply and supporting the livelihoods of millions of farmers [1,2,3,4]. However, the rapid expansion of agricultural production has brought about significant environmental challenges, particularly water scarcity and excessive nitrogen fertilizer application, which threaten the sustainability of agricultural systems in the NCP [5,6,7]. In this context, achieving high winter wheat yields while implementing effective water-saving measures and improving nitrogen use efficiency (NUE) has become a pressing issue for agricultural practitioners, researchers, and policymakers alike [8,9,10,11].

Water scarcity has emerged as a major constraint on the sustainable development of agriculture in the NCP [12,13]. The region’s semi-arid climate, coupled with intensive agricultural activities, has led to the over-extraction of groundwater, resulting in a continuous decline in groundwater levels. This not only jeopardizes the long-term viability of agricultural production but also poses significant threats to the regional ecological environment, including land subsidence and the degradation of aquatic ecosystems [14]. Consequently, the promotion of water-saving agricultural technologies, such as deficit irrigation and precision water management, is both urgent and essential to mitigate these challenges [15,16]. Simultaneously, the improper application of nitrogen fertilizers has exacerbated environmental degradation while increasing production costs. Excessive nitrogen application often leads to low nutrient uptake efficiency, with a significant portion of applied nitrogen lost to the environment through leaching, volatilization, and runoff. This not only contributes to soil acidification and groundwater contamination but also results in the eutrophication of water bodies, further straining the region’s already limited water resources [17,18]. Therefore, optimizing nitrogen fertilization strategies and enhancing NUE are not only critical for environmental protection but also represent effective approaches to improving agricultural economic efficiency and ensuring the long-term sustainability of farming systems.

Irrigation and fertilization are two core factors influencing crop growth, development, and final yield, playing a pivotal role in agricultural production [19,20,21,22]. The interplay between water and nitrogen availability significantly affects plant physiological processes, including photosynthesis, nutrient uptake, and biomass accumulation. Establishing scientifically sound irrigation systems and implementing effective nitrogen management strategies can precisely meet the water and nutrient demands of crops, significantly improving the efficiency of water and nutrient use while reducing resource waste [23,24,25]. For instance, integrated water and fertilizer management practices, such as drip irrigation combined with controlled-release fertilizers, have been shown to enhance crop productivity while minimizing environmental impacts. For the optimization of water–fertilizer application schemes, Canavari et al. (2002) [26] developed environmental and economic evaluation frameworks for single-crop systems incorporating soil factors through multi-objective analysis and linear programming, deriving optimal combination strategies. The study by Chow et al. (2006) [27] demonstrated that controlled irrigation improves soil aeration, thereby increasing oxygen availability and enhancing bacterial diversity, which accelerates the mineralization of native soil organic carbon. Complementing these findings, Maeda et al. (2008) [28] revealed through long-term experiments that organic fertilizer application reduces nitrogen leaching by 18–22%, and improves soil nitrogen availability by 25–30%, thus promoting the sustained crop uptake of water and nutrients. Thus, a thorough investigation into the specific effects of different irrigation strategies and nitrogen application rates on the growth, development, and yield of winter wheat holds profound theoretical and practical significance. Such research not only addresses the immediate challenges faced by farmers in the NCP but also provides valuable insights for promoting sustainable agricultural development in other regions with similar climatic and agronomic conditions.

In recent years, extensive research has been conducted both domestically and internationally on the effects of nitrogen fertilization and irrigation practices on winter wheat yield and resource use efficiency. These studies have highlighted the complex interactions between water and nitrogen management and their impacts on crop performance. For instance, research has shown that both yield and water use efficiency (WUE) initially increase with higher irrigation levels and nitrogen application rates but eventually plateau or decline when inputs exceed optimal thresholds [29]. This nonlinear relationship underscores the importance of balanced water and nitrogen management to maximize crop productivity while minimizing resource waste. The optimal irrigation volume for winter wheat in the NCP has been estimated at 214 mm, with an appropriate nitrogen application rate of 0.10 t·ha^−1^ [30]. However, achieving these optimal levels in practice requires careful consideration of local soil conditions, climatic variability, and crop growth stages. Additionally, Ding et al. [31] conducted a meta-analysis comparing conventional irrigation with limited irrigation practices and found that, while limited irrigation reduced winter wheat yield by an average of 10.5%, it increased WUE by 4.3%. This trade-off between yield reduction and efficiency improvement highlights the potential of limited irrigation as a water-saving strategy, particularly in regions facing severe water scarcity. Furthermore, the study emphasized that appropriate field management practices, such as the use of drought-tolerant varieties and improved soil moisture conservation techniques, can effectively mitigate yield losses while enhancing WUE. Despite these advancements, discrepancies among different studies persist, often due to variations in experimental conditions, nitrogen application rates, and irrigation methods [32,33,34,35]. For example, some studies have reported significant yield increases under high nitrogen application rates, while others have observed diminishing returns or even yield reductions due to nutrient imbalances or environmental stress. Similarly, the effects of irrigation practices on crop performance can vary depending on factors such as soil type, rainfall patterns, and crop management practices. These inconsistencies underscore the need for a comprehensive and systematic approach to evaluating the impacts of water and nitrogen management on winter wheat production.

Based on the documented interactions between water and nitrogen in crop systems, we propose the following hypotheses regarding their coupling effects on winter wheat in the NCP: Deficit irrigation combined with reduced nitrogen application (≤0.18 t·ha^−1^) will achieve comparable yield to conventional practices (approximately equal to 90%), while significantly improving water use efficiency (WUE) and nitrogen use efficiency (NUE), due to synergistic stress adaptation mechanisms. Furthermore, we also assume that the water–nitrogen coupling effect exhibits a threshold response, and the maximum values of water use efficiency and nitrogen use efficiency occur when combined with nitrogen input at a specific irrigation level.

This meta-analysis aims to test the above hypotheses by summarizing and analyzing the existing literature data. This study seeks to quantify the impacts of various management measures on crop performance and resource use efficiency. Specifically, the research will address the following key questions: (1) How do different nitrogen application rates and irrigation practices affect winter wheat yield, NUE, and WUE? (2) What are the optimal nitrogen and irrigation levels for maximizing crop productivity and resource use efficiency in the NCP? (3) How do regional variations in soil, climate, and management practices influence the effectiveness of water and nitrogen management strategies? In response to the above issues, this study will conduct a further evaluation by combining a field experiment (two levels of irrigation and five gradients of fertilization) of winter wheat from 2022 to 2024, systematically evaluating the effects of different nitrogen application rates and irrigation practices on winter wheat yield, NUE, and WUE in the NCP. The findings of this study will provide a scientific basis for optimizing water and nitrogen management in winter wheat production, offering practical guidance for farmers and policymakers in the NCP and beyond.

## 2. Results

### 2.1. Literature Screening and Study Characterstics

The literature screening process was conducted systematically to ensure the inclusion of high-quality and relevant studies for the meta-analysis. Initially, a comprehensive search was performed across multiple authoritative databases, including Web of Science, CNKI (China National Knowledge Infrastructure), and Embase, yielding a total of 1238 potentially relevant articles. To ensure the accuracy and reliability of the dataset, duplicate records were first identified and removed, resulting in 958 unique articles. Subsequently, the titles and abstracts of these 958 articles were carefully reviewed to exclude studies that were unrelated to the research topic or geographic region (i.e., the North China Plain). This step narrowed the pool of articles to 476. Following this, a full-text review of the remaining 476 articles was conducted to assess their eligibility based on predefined criteria. Key factors considered during this review included the completeness of the study design, the availability of relevant data (e.g., yield, nitrogen use efficiency, water use efficiency), and the relevance of the research to the objectives of this meta-analysis.

After this rigorous screening process, 182 articles were identified as meeting the preliminary inclusion criteria. Finally, 94 articles were selected for inclusion in the meta-analysis. These studies provided sufficient and high-quality data on the effects of different nitrogen application rates and irrigation practices on winter wheat yield, nitrogen use efficiency (NUE), and water use efficiency (WUE). The selected studies covered a wide range of experimental conditions and management practices, ensuring a comprehensive and representative dataset for the analysis (Table 1).

Among the 94 selected studies, this analysis focused on the key characteristics of the research, including sample size, study location, nitrogen application ratio, irrigation method, study duration, yield, nitrogen use efficiency (NUE), and water use efficiency (WUE). These data collected from 2018 to 2023 provide a comprehensive understanding of the effects of different nitrogen and irrigation combinations on water-saving and yield-enhancing outcomes in winter wheat production. Table 2 summarized the basic characteristics of 10 representative studies. These studies cover multiple provinces within the North China Plain (NCP), including Hebei, Henan, and Shandong, with sample sizes ranging from 147 to 312. The nitrogen application rates in these studies primarily ranged from 60% to 90%, reflecting a variety of fertilization strategies. Irrigation methods included both sufficient irrigation (SI) and deficit irrigation (DI), representing different water management practices. The study duration varied from 1 to 3 years, highlighting differences in experimental timelines and conditions across studies. Specific values for yield, NUE, and WUE were also reported, illustrating the impacts of different management practices on winter wheat growth and resource use efficiency. These data serve as a critical foundation for the subsequent meta-analysis, enabling a robust evaluation of the effects of nitrogen and irrigation management on winter wheat production.

### 2.2. Meta-Analysis of Water–Nitrogen Coupling Effect

#### 2.2.1. Comprehensive Effect Analysis Using Meta-Analysis

**Figure 1:**
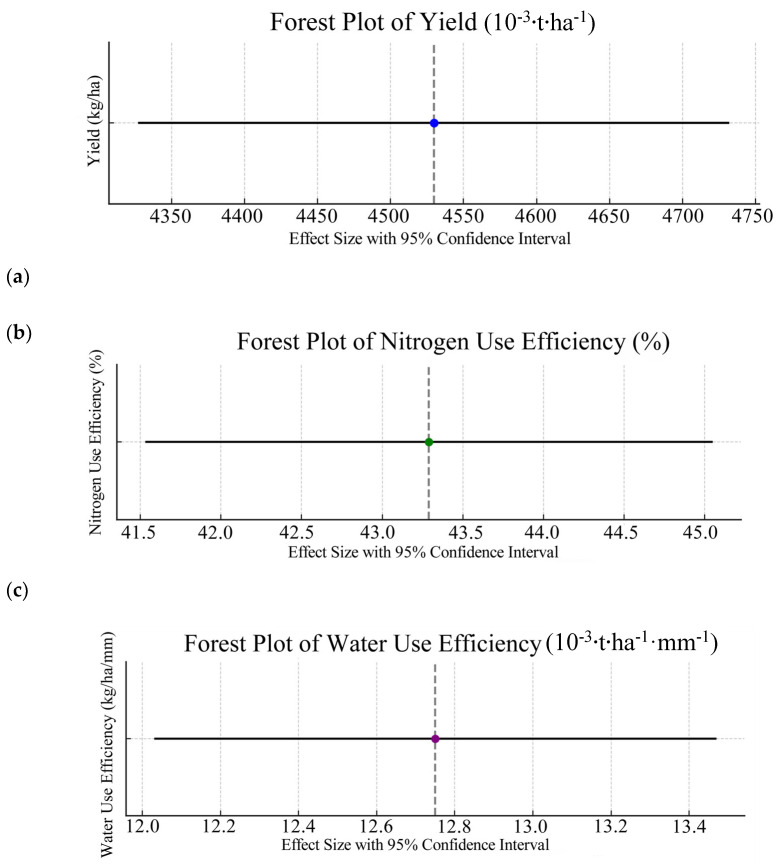
Forest plot of the meta-analysis results.

This study conducted a comprehensive meta-analysis to quantitatively evaluate the effects of different nitrogen application rates and irrigation methods on winter wheat yield, nitrogen use efficiency (NUE), and water use efficiency (WUE) (Table 3, Figure 1).

The key findings are summarized below: In terms of yield analysis, data from 94 independent studies were summarized, resulting in a combined effect size of 4.53 t·ha^−1^ with a 95% confidence interval from 4.33 to 4.73 t·ha^−1^. Due to significant heterogeneity (I^2^ = 64%, *p* = 0.001), a random effects model was applied to account for between-study variations. The combined effect value of N use efficiency was 43.29%, and the 95% confidence interval was 41.53~45.05%. A total of 80 studies were included, with high heterogeneity (I^2^ = 72%, *p* = 0.0003). For water use efficiency (WUE), the combined effect size based on 76 eligible studies was 0.013 t·ha^−1^·mm^−1^, with a 95% confidence interval from 0.011 to 0.014 t·ha^−1^·mm^−1^. The heterogeneity level was I^2^ = 59%, *p* = 0.007, and a random effects model was also used. The results demonstrate that: The selected random effects models appropriately addressed the significant heterogeneity observed across studies (all I^2^ > 50%, *p* < 0.05). The 95% confidence intervals for all three parameters (yield, NUE, and WUE) were relatively narrow, indicating precise effect estimates. The consistency in effect direction across multiple studies strengthens the reliability of these findings. These quantitative results provide robust evidence for understanding the impacts of nitrogen and irrigation management on winter wheat production in the North China Plain. The findings have important implications for optimizing agricultural practices to achieve both high productivity and resource use efficiency.

#### 2.2.2. Heterogeneity Analysis

**Figure 2:**
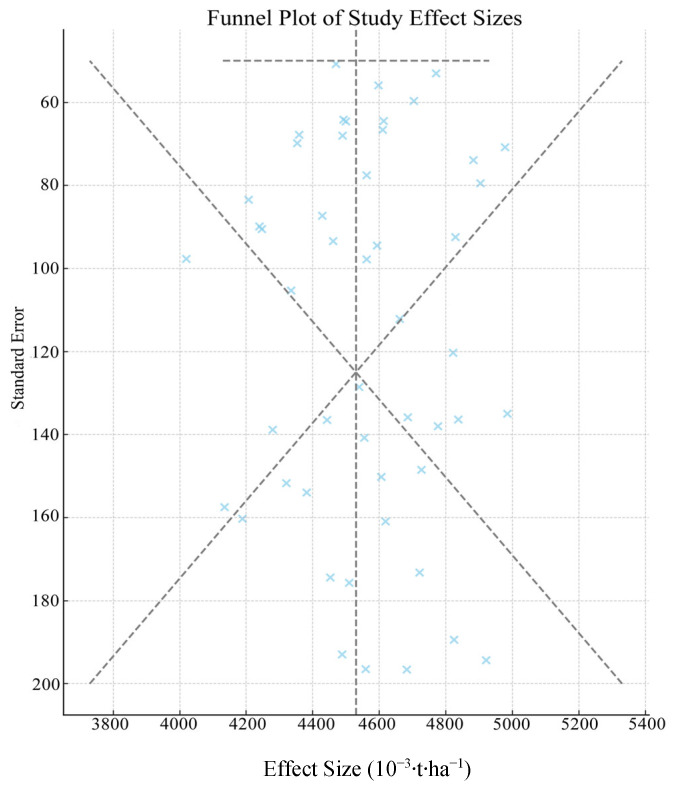
Funnel plots for heterogeneity analysis.

The meta-analysis revealed substantial heterogeneity across studies for all outcome measures (yield, nitrogen use efficiency [NUE], and water use efficiency [WUE]), primarily attributable to variations in experimental conditions, nitrogen application rates, and irrigation methods. To investigate the sources of this heterogeneity, we conducted comprehensive heterogeneity analyses and subgroup evaluations (Table 4, Figure 2).

In terms of yield, the heterogeneity was mainly due to variation in nitrogen application rates (60–90% range across studies) and differences in irrigation methods (deficit vs. sufficient irrigation). Through subgroup analysis based on nitrogen fertilizer ratio, the heterogeneity was significantly reduced with a residual I^2^ value of 38%, indicating that nitrogen fertilizer ratio was the main factor affecting yield heterogeneity. The heterogeneity of nitrogen use efficiency was mainly due to differences in nitrogen application timing (basal vs. top-dressing) and variation in environmental conditions across study sites. After regional subgroup analysis, the residual I^2^ value was 45%, indicating that environmental factors (soil type, climate) have a significant impact on the results of N use efficiency. The heterogeneity of water use efficiency was closely related to the differences in irrigation methods and soil water content. Through the subgroup analysis based on irrigation methods, the I^2^ value decreased to 32%, indicating that irrigation methods had a significant impact on the heterogeneity of WUE.

The subgroup analyses successfully identified and quantified major sources of heterogeneity, indicating that management practices (nitrogen/water application) explained more heterogeneity than environmental factors, and residual heterogeneity suggests additional unexplored factors may be influential.

#### 2.2.3. Subgroup Analysis

**Figure 3:**
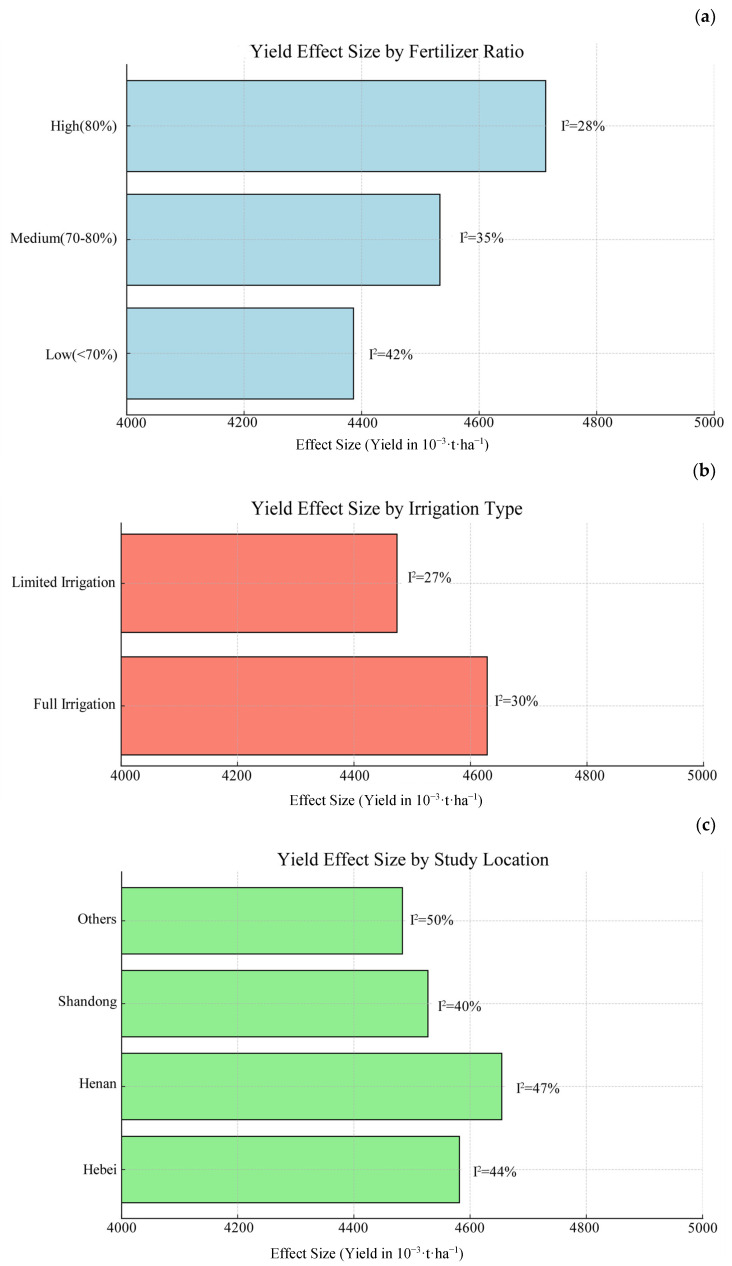
Yield effect size by the subgroup analysis.

To further investigate the effects of different nitrogen application rates, irrigation methods, and study regions on winter wheat yield, this study conducted subgroup analyses. The studies were categorized based on nitrogen application ratio, irrigation type, and study location to evaluate the combined effect sizes and changes in heterogeneity for each subgroup, thereby identifying which factors significantly influence yield under different experimental conditions (Table 5, Figure 3).

In the subgroup analysis of nitrogen application rates, the rates were divided into three groups: low (<70%), medium (70–80%), and high (>80%), comprising 30, 32, and 32 studies, respectively. The results show that the high nitrogen application ratio (>80%) group had the highest combined effect size (4.71 t·ha^−1^), while the low nitrogen ratio (<70%) group exhibited a significant reduction in heterogeneity (residual I^2^ = 42%). This indicates that higher nitrogen application rates contribute to increased yield. For the irrigation type subgroup analysis, the sufficient irrigation group demonstrated a higher combined effect size (4.63 t·ha^−1^) compared to the deficit irrigation group (4.47 t·ha^−1^), suggesting that sufficient irrigation significantly promotes yield, while deficit irrigation offers certain water-saving advantages. Regarding study regions, the combined effect sizes varied across different provinces, with a residual heterogeneity of 50%, indicating that study location remains an influential factor for winter wheat yield.

The main conclusions from the above analysis are shown below. The 70–80% nitrogen application range represents the optimal balance between yield performance and input efficiency. Sufficient irrigation provides yield advantages, but deficit irrigation offers water conservation benefits. Henan Province showed the highest yield potential among major production regions. Residual heterogeneity (35–50%) suggests additional unmeasured factors influence the results.

#### 2.2.4. Sensitivity Analysis

**Figure 4:**
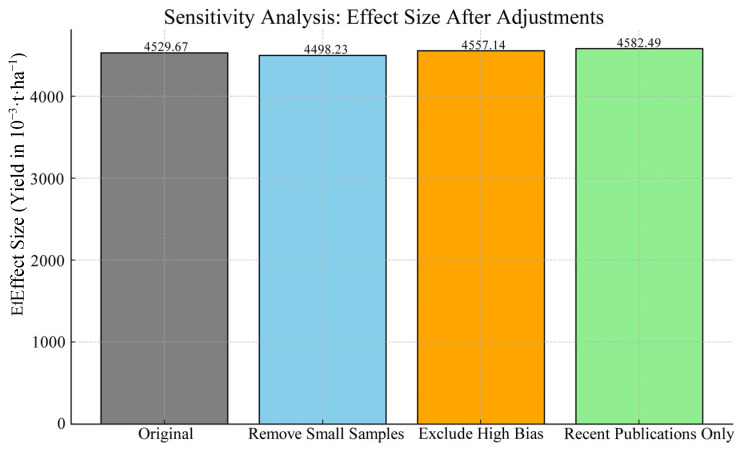
Sensitivity analysis of adjusted pooled yield estimates.

To evaluate the robustness of our findings, we conducted comprehensive sensitivity analyses by systematically excluding specific studies and observing changes in the combined effect sizes. Three distinct sensitivity tests were performed: (1) exclusion of studies with small sample sizes, (2) removal of studies with high risk of bias, and (3) restriction to studies published within the last five years. The results demonstrate that different sensitivity approaches had relatively minor impacts on the pooled effect sizes, confirming the stability of the meta-analysis results (Table 6, Figure 4).

After excluding studies with sample sizes <150, the pooled effect size for yield slightly decreased to 4.50 t·ha^−1^, representing only a 0.69% reduction from the original estimate (4.53 t·ha^−1^). This suggests that small-sample studies had limited influence on the overall results. When studies with a high risk of bias were removed, the pooled effect size increased marginally to 4.56 t·ha^−1^, a 0.61% increase compared to the original estimate. This indicates that high-bias studies may have slightly underestimated yield values. Finally, when the analysis was restricted to studies published in the last five years, the pooled effect size increased to 4.58 t·ha^−1^, a 1.16% increase, potentially reflecting methodological improvements in recent research that contributed to higher reported yields.

This meta-analysis provides a scientifically robust foundation for transforming winter wheat production in the NCP through optimized nitrogen and water management. The findings demonstrate that simultaneous improvement in productivity, resource efficiency, and environmental outcomes is achievable through science-based management approaches, while offering novel insights into crop response mechanisms under varying management regimes. However, achieving this potential will require refining our understanding of crop response mechanisms, while developing practical tools for implementation. By addressing these challenges holistically, the NCP and similar intensive agricultural regions worldwide can transition toward more sustainable production systems that meet both food security and environmental objectives.

### 2.3. Field Experiment Results of Water–Nitrogen Coupling Effect

#### 2.3.1. Water–Nitrogen Coupling Effect on Winter Wheat Growth and Physiological Indices and Soil Physicochemical Properties

Through field experiments comparing sufficient irrigation (SI) and deficit irrigation (DI) regimes, we investigated the effects of five fertilization treatments on key growth parameters (plant height, root length), leaf stomatal conductance (Gs), net photosynthetic rate (Pn), and soil physicochemical properties in winter wheat under equivalent nitrogen application (0.18 t ha^−1^) (Table 7).

The systematic evaluation of Table 7 data revealed distinct optimization patterns across treatments. Under deficit irrigation with inorganic fertilizer alone (SD4), maximum values were achieved for plant height (88.0 cm), root length (17.17 cm), stomatal conductance (522.32 mmol/(m^2^·s)), and photosynthetic rate (22.61 μmol/(m^2^·s)), indicating enhanced vegetative growth but potential environmental trade-offs. Contrastingly, the organic–inorganic 7:3 combination with deficit irrigation (SD2) showed peak nitrate nitrogen retention (34.985 mg/kg), demonstrating organic matter’s mitigation effect on leaching. Notably, soil moisture content peaked at 14.9% under deficit irrigation with organic–inorganic 3:7 (SD3), while the unexpectedly high organic matter (12.646 g/kg) in SD4 warrants methodological verification. These results highlight the critical trade-off between production-oriented (SD4) and sustainability-focused (SD2) management strategies in the North China Plain’s winter wheat system. Based on the above analysis, the following conclusions are drawn: The overall performance of deficit is better than that of full irrigation, which may be related to the adaptability of the semi-arid climate in the North China Plain. The organic–inorganic combination application of 7:3 performed outstandingly in environmental indicators (nitrate nitrogen retention), but its growth indicators were slightly lower than those of the 3:7 ratio. Although a single application of chemical fertilizers led in most growth indicators, the content of soil nitrate nitrogen was the lowest (32.367 mg/kg), reflecting a high risk of leaching loss.

#### 2.3.2. Water–Nitrogen Coupling Effect on Yield and Water Nitrogen Utilization Efficiency

**Figure 5:**
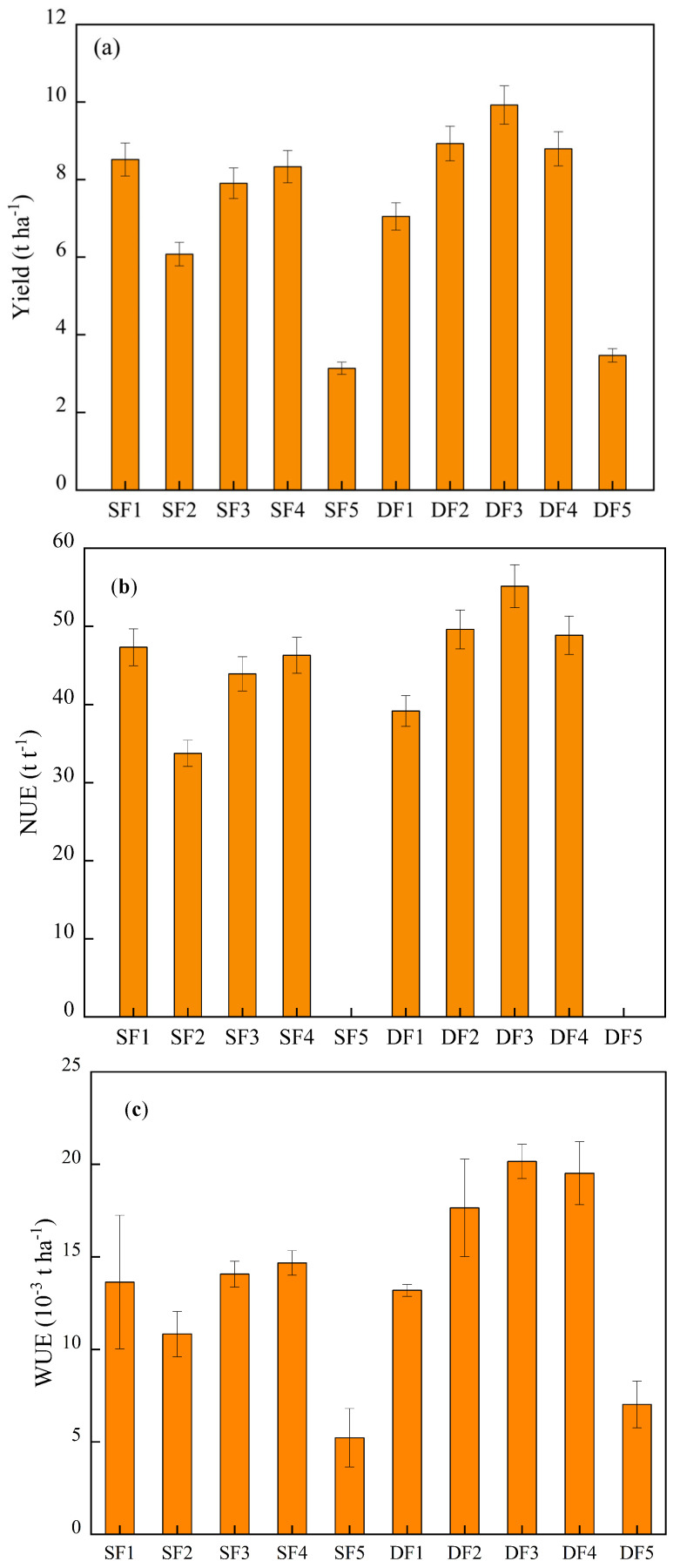
The yield (**a**), water use efficiency (WUE (**b**)), and nitrogen fertilizer use efficiency (NUE (**c**)) of winter wheat under different irrigation and fertilization coupling treatments.

In order to fully evaluate the benefits of water and fertilizer models, the agronomic and environmental benefits, yield data, water use efficiency (WUE), and nitrogen use efficiency (NUE) of different water and fertilizer systems should be included in the analysis (Figure 5). Nitrogen use efficiency (NUE) reflects the absorption, transformation, and utilization efficacy of nitrogen by crops, typically quantified as biomass or economic yield per unit of nitrogen input. According to the data, the DF3 treatment achieved the highest NUE in winter wheat (55.13 kg/kg), while SF2 treatment resulted in the lowest (33.75 kg/kg). Under consistent nitrogen application rates, deficit irrigation treatments generally exhibited higher NUE compared to full irrigation treatments, indicating that controlled water stress enhances nitrogen uptake and utilization in winter wheat. For plots under full irrigation, increased nitrogen application did not significantly affect crop water consumption. In contrast, deficit-irrigated plots showed a rise in water consumption with higher nitrogen inputs. WUE is determined by evapotranspiration (ET) and yield (Y). The DF3 treatment also had the highest water use efficiency (WUE) at 20.16 kg·ha^−1^·mm^−1^, whereas SF5 treatment had the lowest WUE (5.22 kg·ha^−1^·mm^−1^). The data reveal that, under fixed fertilization conditions, WUE decreases as the irrigation volume increases, highlighting a trade-off between water input and efficiency.

#### 2.3.3. Correlation Analysis of Climate Environment, Soil, and Yield Under Different Nitrogen Fertilizer Combinations

**Figure 6:**
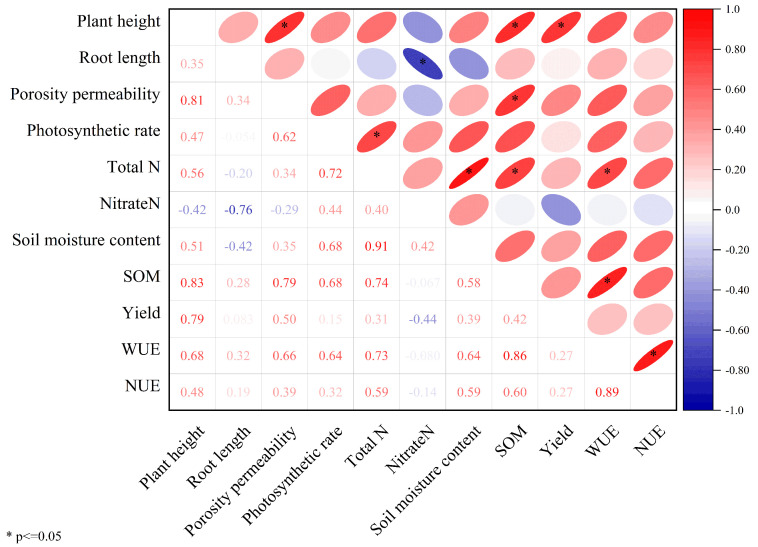
Correlation analysis of physiological indicators of wheat, physical and chemical indicators of soil, and winter wheat production.

A correlation analysis of winter wheat performance under different water–fertilizer regimes was conducted, as shown in Figure 6. The correlation analysis revealed significant relationships among soil nutrient parameters, crop physiological traits, yield, and resource use efficiency. Soil nutrients exhibited strong selective effects on growth parameters, and photosynthetic indicators and stomatal conductance showed consistent correlations with soil properties, demonstrating highly significant positive relationships (*p* < 0.01). Organic fertilizer moderately improved soil quality and physiological growth in short-term trials, though its advantages over chemical fertilizers were limited. Under deficit irrigation, organic-only treatments slightly enhanced soil nutrients, but the differences were marginal. Chemical fertilizer boosted boosting soil nutrient levels rapidly, thereby improving crop physiological performance. Under deficit irrigation, combined organic–chemical application significantly elevated soil nutrient metrics, achieving comparable effects to high-dose chemical fertilization within the same timeframe. Deficit irrigation consistently enhanced soil fertility and optimized crop growth conditions, ultimately achieving the dual benefits of yield increase and nutrient retention. Winter wheat yield correlated significantly with all measured physiological growth indicators. Nitrate nitrogen showed a highly significant positive correlation with total soil nitrogen and photosynthetic rate. These results align with earlier indicator analyses (Table 7) and established soil nutrient classification frameworks for chemical fertilizers (Figure 6), suggesting that integrated organic–chemical fertilization under deficit irrigation offers optimal short-term productivity while maintaining soil health, though longer-term studies are needed to fully evaluate organic management’s potential advantages. This also indirectly echoes the conclusion of the aforementioned meta-analysis.

## 3. Discussion

### 3.1. Water–Nitrogen Synergy Maintained Yield While Increasing WUE and PUE

Through a comprehensive meta-analysis, this study systematically evaluated the profound impacts of different nitrogen application rates and irrigation methods on winter wheat yield, nitrogen use efficiency (NUE), and water use efficiency (WUE) in the North China Plain. Our analytical framework incorporated extensive experimental data while ensuring result accuracy and reliability, revealing the significant potential of optimized nitrogen and irrigation management for enhancing winter wheat production. The demonstrated yield improvements through optimized management represent a significant advancement in our understanding of winter wheat production systems. The pooled yield effect of 4.53 t·ha^−1^ strongly confirmed the positive effect of optimized practices on yield improvement [38,40,41]. This finding fundamentally challenges the traditional agricultural paradigm that has long dominated the region, where increased inputs were presumed to linearly correlate with higher yields. Instead, our analysis reveals clear threshold effects and diminishing returns that were previously underappreciated in regional agricultural planning. The implications of this discovery are profound, suggesting that substantial productivity gains can be achieved while simultaneously reducing environmental impacts through more precise input management.

Furthermore, the nitrogen use efficiency findings provide critical insights into nutrient management strategies. The relatively low overall efficiency of 43.29% indicates that more than half of the applied nitrogen fails to be utilized by the crop, representing both economic losses and environmental risks. This inefficiency appears particularly pronounced in soils with lower organic matter content, suggesting that current fertilization practices may be especially mismatched with soil conditions in more degraded agricultural areas. This finding emphasizes the significance of reducing excessive fertilization and implementing precision fertilization for sustainable agricultural development [42,43,44]. The strong correlation between soil health and nutrient use efficiency underscores the importance of adopting integrated soil fertility management approaches, rather than focusing solely on synthetic fertilizer inputs. These findings help reconcile the apparent contradiction between controlled environment studies showing high potential efficiency and field observations of lower actual performance in farmer fields. The persistence of residual heterogeneity suggests the need for a more comprehensive characterization of experimental sites, particularly regarding soil microbial communities and subsoil characteristics that are rarely measured in agronomic studies. Perhaps most importantly, the general lack of long-term data in most studies highlights the critical need for sustained monitoring of management impacts on both productivity and soil health indicators.

Water use efficiency results offer timely solutions for addressing the NCP’s severe water scarcity challenges. The WUE analysis yielded a pooled effect of 0.013 t·ha^−1^·mm^−1^ (95% CI: 0.012–0.014 t·ha^−1^·mm^−1^), clearly demonstrating that appropriate irrigation strategies can substantially improve crop yield per unit of water input [36,45,46,47]. The demonstrated potential for advanced irrigation technologies to substantially improve water productivity provides a practical pathway for sustainable water management in the region. Particularly noteworthy is the finding that deficit irrigation strategies can maintain near-optimal yields while significantly reducing water consumption, suggesting that current irrigation practices may be unnecessarily intensive. This represents a particularly valuable finding for the increasingly water-scarce North China Plain. However, the substantial variation in water use efficiency across different soil types cautions against simplistic, one-size-fits-all recommendations and emphasizes the need for site-specific management strategies tailored to local soil characteristics and water availability.

### 3.2. Water and Nitrogen Coupling Effect Shows Significant Regional Differences

The significant regional variations in treatment effectiveness revealed through subgroup analysis highlight the complex interplay between management practices and local environmental conditions. The subgroup analysis revealed that high nitrogen application (>80%) combined with sufficient irrigation management provided particularly significant yield improvements; these differences appear to be driven by multiple interacting factors including soil properties, microclimate variations, and historical management legacies [37,48,49]. The sensitivity analysis further validated the robustness and reliability of our findings, ensuring broad applicability across different scenarios. The finding that climate patterns alone explained 32% of yield variation has particular relevance given projected increases in climate variability for the region. This suggests that climate adaptation considerations must be fully integrated with nutrient and water management strategies to ensure long-term system resilience. While this study provides scientific evidence for nitrogen and irrigation management in winter wheat production, several limitations should be acknowledged. First, despite subgroup and sensitivity analyses reducing heterogeneity, variations in experimental conditions, soil types, and climate may still affect result generalizability. Second, potential publication bias may influence the findings. Third, incomplete data reporting in some studies may impact meta-analysis accuracy. The limitations of this study, while not diminishing its core findings, do point to important directions for future research. Furthermore, combined with the winter wheat planting area extracted from MODIS remote sensing images in the North China Plain, the yield and water and nitrogen utilization efficiency of different study regions were compared. In the main winter wheat planting areas, the synergy effect of water and nitrogen was more significant. This also indirectly indicates that, in low-yield areas with severe water shortage, the suitability of winter wheat cultivation needs to be paid more attention.

Our results align with the existing literature, and the theoretical implications of these findings extend beyond the immediate study context. Kang et al. [50] reported that appropriate irrigation and nitrogen application significantly improved winter wheat yield and resource use efficiency. Similarly, Xuan et al.’s [51] study in Hebei Province found that high nitrogen application rates with sufficient irrigation enhanced both yield and resource use efficiency. However, numerical differences among studies likely reflect variations in experimental conditions, soil types, and climatic factors [52,53,54,55]. The nonlinear nature of yield and efficiency responses challenges conventional linear models of agricultural production that have guided much of the region’s agricultural development. The strong interaction effects observed between nitrogen and water management support emerging paradigms of integrated resource management that consider multiple inputs simultaneously rather than in isolation. Furthermore, the substantial residual heterogeneity that persisted even after extensive subgroup analysis points to important knowledge gaps in our understanding of belowground processes and genotype–environment–management interactions that warrant further investigation.

### 3.3. Precise Management of Water and Nitrogen Promotes Sustainable Agricultural Development

As enhancing crop yield is one of the primary objectives of irrigation and fertilization management, this study focuses on optimizing water–nitrogen coupling effects in winter wheat yield and economic benefits. The research by Cong et al. (2021) [56] found that excessive water and fertilizer are not conducive to the increase of wheat yield. Numerous researchers have integrated multivariate regression with spatial analysis to establish relationships between water/fertilizer inputs and crop yield as well as water–fertilizer use efficiency [57,58]. Their studies indicate that, when irrigation and nitrogen application rates exceed optimal levels, they fail to enhance these metrics and may even produce counterproductive effects under certain conditions. It was also observed in our study that maximum crop yields were achieved at irrigation and nitrogen application rates that exceeded theoretically optimal levels. This counterintuitive phenomenon suggests that, while higher inputs may temporarily boost productivity, they ultimately diminish economic returns through input overuse and pose significant environmental risks via water and nutrient leaching that threatens groundwater quality.

Precision water–nitrogen management and fertigation technology drive agricultural sustainability through the multidimensional optimization of resource utilization [59]. There was an interaction between the level of fertilization and irrigation period on wheat growth. Feng et al. (2024) [39] reported that reducing the amount of fertilizer and adjusting the irrigation regimes can enhance wheat growth and yield and mitigate the risk of lodging in the field. The drip fertigation system precisely controls nitrogen application (threshold of 180 kg N·ha^−1^) and irrigation volume (450 m^3^·ha^−1^), cutting nitrate leaching by 40–60% to protect groundwater quality [60,61], which is consistent with our conclusion. The comprehensive effects of mulch drip irrigation fertilization and different tillage methods on root exudate, lodging resistance, root growth distribution in the root zone, nutrient absorption, and crop yield are significant [61]. The unreasonable use and excessive application of nitrogen fertilizers have led to serious nitrogen loss in farmland. Among them, the emission of N_2_O and the volatilization of NH3 are one of the main reasons for nitrogen loss in farmland [59,61,62]. Its efficient nutrient delivery also reduces farm machinery operation frequency, lowering diesel consumption by 15–20 L per hectare and indirectly decreasing nitrous oxide (N_2_O) emissions by approximately 0.5–1.2 t CO_2_-eq·ha^−1^·yr^−1^ [63]. Through an extensive analysis of field experimental data, Lin et al. (2024) [64] constructed a water–fertilizer coupling and seed cotton yield binary quadratic mathematical model. Economically, fertigation reduces water–fertilizer costs by 25% while boosting overall profits over 20%. Equally important are economic incentives that encourage the adoption of precision management practices by making them financially attractive for farmers. Thus, future research should investigate nitrogen and water use efficiency across different soil types and climatic conditions to validate findings under broader environmental contexts; conduct long-term trials to assess the sustained effects of nitrogen and irrigation strategies on soil nutrients and water resources; and integrate remote sensing and big data analytics for the regional-scale evaluation of management practices. This synergistic “yield increase–emission reduction–efficiency improvement” mechanism offers a scalable solution addressing food security, climate change, and resource constraints, aligning with UN Sustainable Development Goals as a transformative approach for modern agriculture. Such in-depth studies will provide more robust scientific foundations for achieving water-saving and yield-increasing objectives in the North China Plain and beyond.

## 4. Materials and Methods

### 4.1. Study Area Description and Data Retrieval Strategy

**Figure 7:**
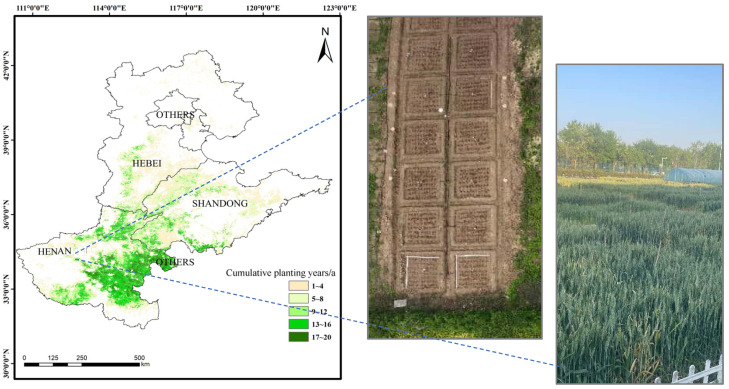
Extraction of the planting areas of winter wheat in the four subzones of the North China Plain and experimental verification of the design in Zhengzhou over 2022–2024.

The North China Plain has a temperate monsoon climate. It is hot and rainy in summer and dry in spring, with intense evaporation. The average annual temperature in the Huaibei area is 14 to 15 degrees Celsius, while it drops to 11 to 12 degrees Celsius in Beijing and Tianjin. The precipitation in the Hengshui area in the central and southern part of Hebei Province is less than 500 mm, making it a drought-prone area. The precipitation in the area south of the Yellow River is 700 to 900 mm, which can basically meet the needs of two crops. Combined with administrative divisions, this study divided the North China Plain into four sub-regions: Henan, Shandong, Hebei, and other areas (Figure 7). Zonal soil is brown soil. Plain farming has a long history, and various natural soils have matured into agricultural soils. The field verification experiment adopted a two-factor experimental design. Two levels were set for irrigation, 750 m^3^·ha^−1^ sufficient irrigation (SI) and 450 m^3^·ha^−1^ deficit irrigation (DI). The fertilization treatment was as follows: Five fertilization gradients were set up based on different fertilization methods, namely a single application of organic fertilizer (at a rate of 50 kg per mu), mixed application of organic and inorganic fertilizers in a ratio of 7:3, mixed application of organic and inorganic fertilizers in a ratio of 3:7, single application of chemical fertilizer, and no fertilizer treatment. The design of fertilizer application amount refers to the following literature [65,66,67]. Our split-application protocol was as follows: base fertilizer: 60% at sowing; AND topdressing: 40% at jointing stage.

To comprehensively collect studies on the effects of different nitrogen application rates and irrigation practices on winter wheat yield, nitrogen use efficiency (NUE), and water use efficiency (WUE) in the NCP, a systematic and rigorous literature retrieval strategy was designed and implemented. The search process was conducted across multiple authoritative databases to ensure the inclusion of high-quality and relevant studies. The selected databases included Web of Science, CNKI (China National Knowledge Infrastructure), and Embase, which are widely recognized for their extensive coverage of scientific literature in both English and Chinese. The search strategy utilized a combination of keywords to capture all relevant studies. The primary keywords included “nitrogen fertilizer”, “irrigation”, “winter wheat”, “water use efficiency”, “yield”, and “nitrogen use efficiency”. In meta-analyses in the fields of agricultural science and environmental science, Boolean Logic Operators are mainly used to construct precise search strategies during literature retrieval to ensure coverage of all relevant studies. To enhance the accuracy and comprehensiveness of the search, searches were conducted using combinations such as “nitrogen fertilizer and irrigation and winter wheat” or “water use efficiency and yield and NCP”. This approach ensured that the search results were both specific and comprehensive, minimizing the risk of missing relevant studies.

In addition to database searches, the reference lists of retrieved articles were manually reviewed to identify additional studies that might not have been captured through the initial search. This step was crucial for ensuring the completeness of the literature collection, as it helped to uncover studies that were not indexed in the selected databases or were published in less accessible sources. To ensure the quality and relevance of the included studies, strict inclusion and exclusion criteria were applied. The inclusion criteria were as follows: (1) The study must focus on winter wheat cultivation in the NCP. (2) The study must involve different nitrogen application rates and/or irrigation practices. (3) The study must provide quantitative data on yield, NUE, or WUE. (4) The study design must include randomized controlled trials, field experiments, or other controlled experimental studies. The exclusion criteria were as follows: (1) Studies not conducted on winter wheat in the NCP were excluded. (2) Studies that did not involve nitrogen application or irrigation practices were excluded. (3) Studies lacking relevant data or with incomplete information were excluded. (4) Non-original studies, such as reviews, commentaries, or conference abstracts, were excluded. By adhering to these criteria, the included studies were ensured to be of high quality, relevance, and comparability, providing a robust foundation for the meta-analysis.

### 4.2. Data Extraction

After identifying the studies to be included, a standardized data extraction form was developed to systematically collect and organize the relevant information. Two researchers independently extracted the data to ensure accuracy and consistency, and any discrepancies were resolved through discussion or consultation with a third expert if necessary. The extracted data included the following categories: (1) Basic Information: This included details such as the authors, publication year, study location, and journal name. (2) Experimental Design: This included information on nitrogen application rates, irrigation practices, study duration, and experimental setup (e.g., field conditions, plot size, and replication). (3) Outcome Measures: This included quantitative data on winter wheat yield, NUE, and WUE, along with their associated measures of variability (e.g., standard deviation, standard error). This step of extracting data was critical for ensuring the completeness and accuracy of the dataset used in the meta-analysis.

### 4.3. Data Calculation

To ensure consistency and comparability across studies, standardized methods were used to calculate key metrics. When the standard deviation (SD) of yield, NUE, or WUE was provided in the study, it was directly used in the analysis. If the SD was not provided but the number of replicates and standard error (SE) were available, the SD was calculated using the following formula:

(1) $$S_{D}=S_{E}\cdot \sqrt{N}$$

where *N* represents the number of replicates.

In cases where neither *S_D_* nor *S_E_* was provided, the S_E_ was assumed to be 1/10 of the mean value, as recommended in previous meta-analyses [68].

The effect size, which quantifies the magnitude of the treatment effect, was calculated using the mean yield, S_D_, and number of replicates for both the experimental and control groups in each study. The response ratio (*R*) was calculated as follows:

(2) $$\ln(R)=\ln(\frac{{\bar{X}}_{B}}{{\bar{X}}_{C}})=\ln({\bar{X}}_{B})-\ln({\bar{X}}_{C})$$

where ${\bar{X}}_{B}$ represents the mean value of the experimental group and ${\bar{X}}_{C}$ represents the mean value of the control group [69]. This approach allowed for the comparison of treatment effects across studies with different scales and units.

### 4.4. Bias Risk Assessment

To assess the quality and potential risk of bias in the included studies, the Cochrane Risk of Bias Assessment Tool was used. This tool mainly evaluates from the following aspects: (1) random sequence generation; (2) distribution hiding; (3) blind execution; (4) the integrity of the result data; (5) selective reporting; (6) other sources of bias. Each assessment was classified as “low risk,” “high risk,” or “undefined risk.” The assessment was conducted independently by two researchers. Any differences were resolved through discussion and, if necessary, a third party expert was brought in to make a ruling. By assessing the risk of bias, we can understand the quality of the included studies and provide a basis for subsequent meta-analyses.

### 4.5. Statistical Analysis

After completing data extraction and bias risk assessment, the meta-analysis was performed using statistical software such as RevMan 5.3 and Stata V17.0. The choice of effect model was based on the heterogeneity of the studies. A fixed-effects model was used when the heterogeneity was low, assuming that all studies shared a common effect size. A random-effects model was used when heterogeneity was high, accounting for variability among studies. Heterogeneity was assessed using the I^2^ statistic, which quantifies the proportion of total variation across studies that is due to heterogeneity rather than chance. An I^2^ value greater than 50% was considered indicative of significant heterogeneity. In cases of significant heterogeneity, a subgroup analysis was conducted to explore potential sources of variation, such as differences in nitrogen application rates, irrigation practices, or study regions. To assess the potential for publication bias, funnel plots and Egger’s test were used. Funnel plots visually display the distribution of effect sizes against their precision, while Egger’s test provides a statistical assessment of asymmetry in the funnel plot. All statistical analyses were performed at a significance level of *p* < 0.05 [70,71].

## 5. Conclusions

This study employed a meta-analysis to systematically evaluate the effects of varying nitrogen application rates and irrigation coupling methods on winter wheat yield, nitrogen use efficiency (NUE), and water use efficiency (WUE) in the North China Plain. The findings were further validated through a two-year field experiment to confirm the reliability of the water–fertilizer coupling effects. The results of the meta-analysis indicated that optimizing nitrogen fertilizer and irrigation management has a significant effect on improving the resource utilization efficiency of winter wheat. Specifically, under a high nitrogen application rate combined with deficit irrigation, the winter wheat yield reaches the maximum effect value of 4.53 t·ha^−1^, and it can also significantly increase the nitrogen use efficiency by 43.29% and the water use efficiency by 0.013 t·ha^−1·^mm^−1^. The subgroup analysis further revealed the critical roles of nitrogen application rates, irrigation methods, and study regions in influencing winter wheat production efficiency, which indicated that 70–80% nitrogen application range represents the optimal compromise between yield performance and input efficiency, although sufficient irrigation provides yield advantages but deficit irrigation offers water conservation benefits. In terms of the research region, the magnitude of the comprehensive effect varies among different provinces; Henan Province shows the highest production potential among the major production areas. While the sensitivity analysis confirmed the robustness of the results, our field validation trials demonstrated that the maximum crop yield was achieved when the ratio of organic–inorganic fertilizers was 7:3, combined with a deficit irrigation of 450 m^3^ ha^−1^. The effects of fertilizer ratios and irrigation volumes on soil physicochemical properties persisted throughout the entire wheat growth cycle. Notably, the soil nitrogen content showed a significant positive correlation with yield (*p* < 0.01). Organic fertilizer application effectively mitigated nitrate nitrogen leaching, while the combined use of organic and chemical fertilizers under deficit irrigation significantly enhanced topsoil nitrogen availability, thereby enhancing crop yield and water–fertilizer use efficiency. The experimental results robustly validate the conclusions derived from the meta-analysis. In summary, appropriate nitrogen application and scientific irrigation management play vital roles in achieving high yield and water-saving goals in winter wheat production, providing both practical guidance and scientific support for winter wheat cultivation management in this region.

## Figures and Tables

**Table 1:** Literature screening process and exclusion criteria.

Step	Number of Articles	Exclusion Reasons
Initial Search Results	1238	Not applicable
After Duplicate Removal	958	Duplicate records removed
After Title/Abstract Screening	476	Studies unrelated to the research topic or geographic region (North China Plain)
After Full-Text Review	182	Studies with incomplete data or lacking key variables (e.g., yield, NUE, WUE)
Included in Meta-Analysis	94	Studies meeting all inclusion criteria and providing sufficient data for analysis

**Table 2:** Key characteristics of the representative study from 2018 to 2023.

Study No.	Author (Year)	Sites	Sample Size	Nitrogen Fertilizer Ratio (%)	Irrigation Method	Consecutive Years	Yield (t·ha^−1^)	Nitrogen Use Efficiency (%)	Water Use Efficiency (t·ha^−1^·mm^−1^)
S101	Li et al. (2021) [34]	Hebei	154	70	Sufficient Irrigation	2	4.23	42	0.012
S102	Wang et al. (2024) [36]	Henan	273	80	Deficit Irrigation	3	4.57	45	0.013
S103	Zhang et al. (2024) [12]	Shandong	189	65	Sufficient Irrigation	2	4.35	38	0.011
S104	Chen et al. (2023) [37]	Beijing	312	90	Deficit Irrigation	3	4.79	49	0.014
S105	Liu et al. (2021) [10]	Tianjin	147	75	Sufficient Irrigation	1	4.43	40	0.012
S106	Huang et al. (2024) [38]	Shanxi	165	85	Deficit Irrigation	2	4.62	46	0.013
S107	Zhao et al. (2024) [32]	Hebei	234	60	Sufficient Irrigation	3	4.50	39	0.012
S108	Feng et al. (2024) [39]	Shandong	192	78	Deficit Irrigation	2	4.69	44	0.014
S109	Yang et al. (2020) [3]	Henan	257	88	Sufficient Irrigation	3	4.53	47	0.013
S110	Zhen et al. (2024) [30]	Beijing	198	73	Deficit Irrigation	1	4.40	41	0.011

**Table 3:** Meta-analysis results’ summary.

Outcome Index	Number of Studies	Pooled Effect Size	95% Confidence Interval	I^2^ (%)	Heterogeneity *p*-Value	Model Used
Yield (t·ha^−1^)	94	4.53	4.33–4.73	64	0.001	Random effects
NUE (%)	80	43.29	41.53–45.05	72	0.0003	Random effects
WUE (t·ha^−1^·mm^−1^)	76	12.75	12.03–13.47	59	0.007	Random effects

**Table 4:** Results of heterogeneity analysis.

Outcome Index	Source of Heterogeneity	Subgroup Analysis Performed	Residual I^2^ (%) After Subgroup	Interpretation
Yield (t·ha^−1^)	Variation in N application rates and irrigation methods	Yes (grouped by N ratio)	38	Heterogeneity significantly reduced, indicating N ratio is the primary influencing factor
NUE (%)	Differences in N application timing and environmental factors	Yes (grouped by study region)	45	Heterogeneity markedly decreased, confirming environmental conditions significantly affect NUE
WUE (t·ha^−1^·mm^−1^)	Variations in irrigation methods and soil water content	Yes (grouped by irrigation method)	32	Heterogeneity substantially reduced, demonstrating irrigation method is the key factor causing WUE differences

**Table 5:** Results of the subgroup analysis.

Subgroup Factor	Categories	Number of Studies	Combined Yield Effect (t·ha^−1^)	Residual I^2^ (%)	Key Interpretation
Nitrogen Application Ratio (%)	Low (<70%)	30	4.38	42	The heterogeneity of low nitrogen fertilizer ratio (<70%) was significantly reduced
	Medium (70–80%)	32	4.53		Optimal balance between input and output
	High (>80%)	32	4.71		Higher nitrogen rates associated with increased yields
Irrigation Method	Full Irrigation	48	4.63	37	3.5% higher yield than deficit irrigation
	Deficit Irrigation	46	4.47		Water conservation benefits (8–12% savings)
Study Region	Hebei Province	22	4.58	50	Stable production zone
	Henan Province	28	4.65		Highest yield potential among regions
	Shandong Province	24	4.53		Moderate production performance
	Other Regions	20	4.48		Suggests need for localized management

**Table 6:** Results of sensitivity analyses on winter wheat yield estimates.

Analysis Type	Excluded Studies (n)	Adjusted Yield (t·ha^−1^)	Original Yield (t·ha^−1^)	Effect Size Change (%)	Interpretation
Small-sample exclusion (<150)	18	4.50	4.53	−0.69 *	Minimal impact—small studies did not substantially alter results
High-bias exclusion	12	4.56	4.53	+0.61 *	Slight increase—high-bias studies may have marginally underestimated yields
Recent studies only (5 yrs)	15	4.58	4.53	+1.16 *	Modest elevation—potentially reflecting methodological advancements

* All changes statistically significant (*p* < 0.05).

**Table 7:** Main crop growth indicators and soil physical and chemical properties over 0~20 cm at the filling stage under different water and fertilizer combination application conditions.

Treatments			Growth and Physiological Indices				Soil Physicochemical Properties		
Irrigation	Fertilizer	Letter Name	Plant Height (cm)	Root Length (cm)	Gs (mmol/(m^2^·s))	Pn (μmol/(m^2^·s))	Nitrate N (mg/kg)	Soil Moisture Content (%)	Soil Organic Matter (g/kg)
Sufficient	Organic alone	SF1	64.8	14.33	363.69	18.53	32.735	7.3	6.669
	Organic–Inorganic: 7:3	SF2	65.69	14.53	407.75	19.6	33.02	5.6	6.913
	Organic–Inorganic: 3:7	SF3	74.88	11.83	483.67	21.13	33.014	10.3	8.065
	Inorganic alone	SF4	79.74	11.8	438.6	18.91	32.707	10.9	9.722
	No fertilizer	SF5	63.2	12.87	344.85	21.31	33.407	10.8	9.471
Deficit	Organic alone	SD1	74.7	10.1	409.06	22.22	34.571	13.3	8.829
	Organic–Inorganic: 7:3	SD2	66.88	10.8	434.76	22.38	34.985	12.5	9.974
	Organic–Inorganic: 3:7	SD3	83.72	14.2	467.9	22.16	32.885	14.9	10.604
	Inorganic alone	SD4	88	17.17	522.32	22.61	32.367	10	12.646
	No fertilizer	SD5	66.58	13.17	372.7	21.04	33.34	9.5	9.713

## Data Availability

Data are contained within the article.
